# Public pensions and unmet medical need among older people: cross-national analysis of 16 European countries, 2004–2010

**DOI:** 10.1136/jech-2015-206257

**Published:** 2016-12-13

**Authors:** Aaron Reeves, Martin McKee, Johan Mackenbach, Margaret Whitehead, David Stuckler

**Affiliations:** 1 International Inequalities Institute, London School of Economics and Political Science, UK; 2 Department of Sociology, University of Oxford, Oxford, UK; 3 Department of Public Health and Policy, LSHTM, London, UK; 4 Department of Public Health, Erasmus MC, Rotterdam, Netherlands; 5 Department of Public Health and Policy, University of Liverpool, Liverpool, UK

**Keywords:** ACCESS TO HLTH CARE, POLICY, ELDERLY

## Abstract

**Background:**

Since the onset of the Great Recession in Europe, unmet need for medical care has been increasing, especially in persons aged 65 or older. It is possible that public pensions buffer access to healthcare in older persons during times of economic crisis, but to our knowledge, this has not been tested empirically in Europe.

**Methods:**

We integrated panel data on 16 European countries for years 2004–2010 with indicators of public pension, unemployment insurance and sickness insurance entitlement from the Comparative Welfare Entitlements Dataset and unmet need (due to cost) prevalence rates from EuroStat 2014 edition. Using country-level fixed-effects regression models, we evaluate whether greater public pension entitlement, which helps reduce old-age poverty, reduces the prevalence of unmet medical need in older persons and whether it reduces inequalities in unmet medical need across the income distribution.

**Results:**

We found that each 1-unit increase in public pension entitlement is associated with a 1.11 percentage-point decline in unmet medical need due to cost among over 65s (95% CI −0.55 to −1.66). This association is strongest for the lowest income quintile (1.65 percentage points, 95% CI −1.19 to −2.10). Importantly, we found consistent evidence that out-of-pocket payments were linked with greater unmet needs, but that this association was mitigated by greater public pension entitlement (β=−1.21 percentage points, 95% CI −0.37 to −2.06).

**Conclusions:**

Greater public pension entitlement plays a crucial role in reducing inequalities in unmet medical need among older persons, especially in healthcare systems which rely heavily on out-of-pocket payments.

## Introduction

It is commonly assumed that older persons have largely been spared the effects of the Great Recession.^1^ Rising unemployment and stagnating wages—two major consequences of the economic crisis—would not be expected to have directly undermined pensioners' financial security, although there is some evidence that retirees now provide greater financial support to their younger relatives.^2^ Austere welfare measures have been concentrated on working-age adults,^3^ largely for political reasons.

Despite apparently being spared the worst of austerity policies, there is cause for concern. Since the onset of the Great Recession, unmet need for medical care—a comparative measure of healthcare access defined as being unable to obtain care when the person believed it to be medically necessary—has been increasing in many European countries, especially in persons aged 65 or older.^4^ ^5^ This is a matter of concern on health and economic grounds. Increasing unmet medical need, while potentially reducing spending in the short term, might increase future healthcare costs because treatment is postponed.^6^ Moreover, the increase in unmet need is taking place at a time when many European countries are cutting budgets for social care.^1^ ^7^

Given that many European countries have health systems that provide free, or largely free care, especially for their older citizens, what can explain these findings? One possibility is that older people may face barriers to accessing health facilities, for example, because of the cost or availability of transport, especially if public pensions have failed to keep pace with living costs.^8^ Although in recent years public pensions have avoided the brunt of budget cuts, over the last decade, pensioners have actually faced various forms of retrenchment in pension spending in some European countries so that, in 2013, the Organisation for Economic Co-operation and Development (OECD) expressed concern that raising state retirement ages and freezing (and, in some case, cutting) public pensions any further would negatively influence older persons' financial security.^9^ The magnitude of this retrenchment has been obscured, in data on aggregate spending, by the increase in the number of pensioners during this same period, so that total expenditure on public pensions has risen.^3^ Some countries have also altered indexation rules in order to reduce public pension benefits. For example, the Czech Republic, Hungary and Norway no longer index public pensions to wage growth, while Austria, Greece, Portugal and Slovenia have stopped doing so for all but those on the lowest public pensions, in each case leading to real declines. Greece's reforms have been particularly severe. Public pension benefits are now calculated on the basis of average earnings across a worker's lifetime rather than on their final salary, reducing pension benefits by between 5% and 15%. The distributional effect of these reforms vary across countries, with reforms reducing replacement rates among some of the poorest groups in some (the UK) but not all (Italy) countries.^9^ In short, reduced public pension entitlement may reduce the ability to pay for the various costs involved in seeking healthcare and in keeping healthy and staying independent, particularly for those at the bottom of the income distribution.^10–12^

Reduced public pension entitlements may also reduce access to care among those not of pension age. Financial transfers from older people to their children and grandchildren have increased over time and such transfers directly impact health outcomes in some contexts.^2^ ^13^ Cuts to public pensions may therefore also put additional financial pressure on low-wage workers, potentially reducing access to care.^5^

Any such problems have only been compounded by greater user charges, which have increased the cost of pharmaceuticals, outpatient care and accident and emergency (A&E) visits in some countries.^14^ Even where older people are insured, out-of-pocket payments deter them from accessing the care they believe they need.^15^ ^16^ Despite heated debate regarding their utility, user charges have been extended and expanded across Europe.^5^ ^15^ In 2009–2010, user charges for prescription drugs and dentistry in the UK's National Health Service generated around £1 billion a year. Even though exemptions exist for children, older people and people on low incomes, these charges have been described as a ‘mess’ in part because they still create financial barriers to access for these economically vulnerable groups.^15^ ^17^ User charges were increased for inpatient care in France in 2010 and other charges were increased for outpatient prescription drugs and primary care during this same period.^18^ During the financial crisis, countries with higher levels of user charges experienced a sharper decline in health service usage than countries where user charges were lower.^19^

Alternatively, some have argued that unmet medical needs are not a concern for public health, claiming that those who report unmet medical needs are over-users, for whom missing one visit will cause very little harm.^16^ Yet while, in theory, user charges may encourage people to be more discerning in choosing healthcare services,^20^ there is considerable evidence that most are unable to make such complex decisions and so reduce necessary and unnecessary healthcare, particularly low-income patients.^15^ ^21–24^ Although an association between unmet medical need and health is plausible, evidence documenting this relationship is surprisingly scarce and there remains uncertainty regarding whether and how this works. While some studies have been too short to document the health effects of such decisions, there is evidence that substantial rises in unmet medical need can adversely affect health. Longitudinal data over 6 years from the USA found that greater access to care among those insured increased survival chances and reduced the likelihood of transitioning into disability by roughly 30%.^25^ Wang and colleagues find that in 2003, when healthcare usage decreased because of fear of contracting severe acute respiratory syndrome (SARS) when visiting health facilities, mortality from diabetes mellitus and cerebrovascular diseases significantly increased by 8.4% and 6.2%, respectively.^26^ Of course, unmet medical needs may have long-term consequences that are difficult to assess, such as if cancer is undiagnosed.^6^

Given the potential health consequences of rising unmet medical need, we test the hypothesis that greater public pension entitlement reduces unmet medical need in older persons during the Great Recession. This is our primary research question. We also test a number of subhypotheses that the association between public pensions and unmet medical need will be greater (i) for poorest income groups and (ii) in countries where there is a high reliance on out-of-pocket spending. Finally, because greater public pension entitlements also benefit people who are not of pension age,^13^ we test whether public pension entitlements may have spillover effects for access to care among those not of pension age, hypothesising that public pensions will also reduce unmet medical need among working-age populations but to a lesser extent.

### Data and method

We collected data from 16 European countries (Austria, Belgium, Denmark, Finland, France, Germany, Greece, Ireland, Italy, Netherlands, Norway, Portugal, Spain, Sweden, Switzerland and the UK) between 2004 and 2010 from EuroStat and the Comparative Welfare Entitlements Dataset (CWED).^27^ Data on unmet medical need due to cost are from EuroStat 2014 edition, which derive from nationally representative individual-level surveys (EU Statistics on Income and Living Conditions, EU-SILC).^28^ EU-SILC survey participants are asked: ‘Was there any time during the last twelve months when, in your opinion, you personally needed a medical examination or treatment for a health problem but you did not receive it?’.^16^ We measure the proportion of respondents in each country in a given year who answer yes to this question, that is, their medical need was unmet due to cost. We also collated disaggregated measures of unmet need by age and income quintile at the country level from EuroStat. As a falsification test, we examine data on unmet medical need for other reasons (not cost related). All other macroeconomic data, including gross domestic product (GDP) and government health spending per capita, were adjusted for inflation and purchasing power, and taken from EuroStat. Data are missing data for some country-years, in particular measures of unmet medical need by income quintile are not reported for every quintile in some countries.

To measure welfare entitlement, we use the CWED, covering the period 2004–2010. Welfare entitlement is composed of three separate measures, including entitlement to public pensions, sickness benefit and unemployment benefit.^27^ More details on how these measures are calculated are in the documentation for the database.^27^ In this paper, the public pension entitlement indicator is of particular interest. It is calculated by combining country-year observations of (1) minimum income replacement rate of the public pension, (2) the average income replacement rate of all pensions, (3) the expected duration of the pension, (4) the number of years of insurance needed for a standard pension, (5) a measure of the ratio of the proportion of employee to employee-plus-employer contributions for the pension and (6) an estimate of the portion of those above retirement age who are in receipt of a public pension. This measure excludes occupational pensions, except for the nominally private Finnish pension system, and therefore does not capture inequalities in income during retirement. However, it does measure the basic pension level within that country. State pensions will matter more for those at the bottom of the income distribution, which is also where rates of unmet medical need are highest. Measuring pension entitlement in this way (eg, public pension) also allows us to observe whether changes in income due to state pensions reduce inequalities in unmet medical need across income groups. Throughout the study period, most of the variation in public pension entitlement was attributable to changes in the minimum income replacement rate of pensions and, to some extent, the standard replacement rate, suggesting that changes were primarily driven by the amount received. For this measure, a 1-unit change in public pension entitlement is the equivalent of a 10 percentage-point increase in the minimum income replacement rate, something Ireland implemented between 2005 and 2007. Comparable measures of unmet medical need are only available from EuroStat from 2004 onward, via the EU-SILC, and comparable measures of public pension generosity are only available up to 2010 for OECD countries included in the dataset created by Scruggs and colleagues. These constraints define our analytic sample.

To address our research questions, we estimate an ecological model of the association between public pension entitlement and unmet medical need. We do not estimate a multilevel model (including individual-level predictors) because public pension entitlements are largely independent of individual covariates. Further, given that SEs are calculated at the country level, a multilevel model should not substantially alter our findings. We test this assumption as part of our sensitivity tests described below. Thus, our statistical model of the influence of public pension entitlement on unmet medical need is as follows:1
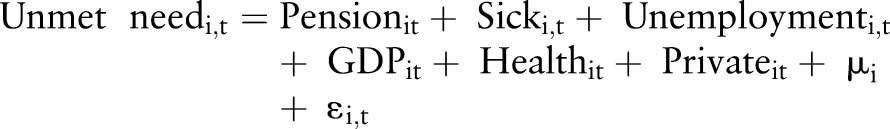
Where i is country and t is year. Unmet need is a vector of unmet medical need indicators by age and by income quintile. In the main models of this paper, we examine the association between public pensions and unmet medical need due to cost. As a falsification test, we examine the association between public pensions and unmet medical need for other reasons. Pension is a measure of public pension entitlement. Sick is a measure of welfare entitlement to those who experience medium-term or long-term sickness and are unable to work, which is available to pensioners in some countries. Unemployment is a measure of welfare entitlement for those who are unemployed, which captures the degree of decommodification in a given society and reflects the strength of the social safety net and is also a proxy for the decommodification of healthcare provision. GDP measures change in GDP per capita over time, adjusted for inflation and purchasing power, capturing the real value of the average level of individual income. Health is a measure of total government health spending per capita, also adjusted for inflation and purchasing power. Finally, Private includes a measure of private pensions from the OECD, which includes mandatory and voluntary contributions, which may predict access to healthcare.^13^ ^29^ ^30^ This indicator is not directly comparable with our measure of public pension entitlement but does capture alternative sources of income for pension citizens.^27^ µ is a country-specific indicator which captures differences between countries that remain relatively stable over time. These so-called fixed-effects models remove between-country differences and examine only the change within countries over time.^31^ ^32^ ε is the error term. Robust standard errors were calculated to address heteroscedasticity, clustered by country to reflect non-independence of sampling within countries over time. In those models where we specifically examine the unmet medical need of older persons (65+), we also include a measure of the proportion of the older population who self-report a chronic illness as this will capture changes in medical need over time. This is also taken from EuroStat and drawn from the EU-SILC data, which asks respondents whether they ‘suffer from any chronic (long-standing) illness or condition’. Finally, to test whether any association between public pensions and unmet medical due to cost is moderated by the extent of out-of-pocket payments, we include a measure of out-of-pocket payments for healthcare (measured as a proportion of GDP) into equation 1 and an interaction term between this measure of out-of-pocket payments and our measure of pension entitlements. Descriptive statistics for these variables are in web appendix 1. All models were estimated using STATA V.12.

10.1136/annrheumdis-2016-210131.supp1 supplementary appendix

## Results

### Does greater public pension entitlement reduce unmet medical need among older persons?

First, we examined the effect of public pension entitlement on those who were over the age of 65 (table 1). In these models, we include a measure of the proportion of the population over the age of 65 that has a chronic illness. Public pension entitlement is closely associated with unmet medical need due to cost. A 1-unit increase in public pension entitlement (the equivalent of 10 percentage-point increase in the minimum income replacement rate) is associated with a 1.11 percentage-point decline in unmet medical need among over 65s (95% CI −0.55 to −1.66). There is no clear association between unmet medical need and sickness insurance entitlement (p=0.55), unemployment insurance entitlement (p=0.57), government expenditure on health (p=0.13) or the proportion of older persons who report living with chronic illnesses (p=0.60). An increase in GDP tends to reduce unmet medical need (b=−0.03, p=0.08), but the association is not significant at the α=0.05 level. Private pensions play an important role in many European countries, and so we include a measure of private pension expenditure (incorporating mandatory and voluntary contributions) as a percentage of GDP from the OECD. However, we find that not only do our results remain qualitatively similar but we also observe that this measure of private pensions is not associated with unmet medical need (web appendix 2). This is consistent with previous work finding that basic pensions (but not earnings-related pensions) reduce premature mortality among the elderly.^10^

**Table 1:** Impact of public pension entitlement on unmet medical need due to cost among older people (65+), 2004–2010

	Unmet medical need due to cost (percentage point) among over 65s					
Covariates	(Model 1)	(Model 2)	(Model 3)	(Model 4)	(Model 5)	(Model 6)
Public pension entitlement	−1.00* (−1.81 to −0.20)	−1.06** (−1.76 to −0.37)	−1.13** (−1.82 to −0.45)	−1.12** (−1.74 to −0.50)	−1.11** (−1.66 to −0.55)	−1.11** (−1.67 to −0.55)
Sickness insurance entitlement		−0.85* (−1.69 to −0.0090)	−0.76 (−1.91 to 0.39)	−0.44 (−1.62 to 0.74)	−0.35 (−1.59 to 0.88)	−0.35 (−1.60 to 0.91)
Unemployment insurance entitlement		0.11 (−0.42 to 0.65)	0.16 (−0.42 to 0.74)	0.12 (−0.41 to 0.65)	0.15 (−0.41 to 0.72)	0.16 (−0.39 to 0.71)
US$100 increase in public health expenditure per capita			0.053 (−0.14 to 0.25)	0.18 (−0.068 to 0.43)	0.20 (−0.067 to 0.46)	0.19 (−0.075 to 0.46)
US$100 increase in GDP per capita				−0.029 (−0.063 to 0.0047)	−0.030 (−0.064 to 0.0039)	−0.030 (−0.065 to 0.0054)
Proportion of older people with a chronic illness (%)					−0.022 (−0.11 to 0.067)	−0.022 (−0.11 to 0.067)
Private pension expenditure (% GDP)						0.035 (−0.77 to 0.84)
Observations	103	103	103	103	103	103
R^2^	0.23	0.26	0.26	0.34	0.35	0.35

*Notes:* Sources: Comparative Welfare Entitlements Dataset, Organisation for Economic Co-operation and Development (OECD) and Eurostat. Expenditure measures are adjusted for inflation and purchasing power parity. All models adjust for country-specific differences that are constant over time. 95% CIs in parentheses.

*p<0.05; **p<0.01.

GDP, gross domestic product.

### Does greater public pension entitlement reduce inequalities in access to healthcare?

Next, we tested whether the benefits of public pension entitlement were greater in older persons who were in the lowest income quintile. The observed relationship was, as anticipated, stronger. As shown in Model 1 in web appendix 3, each 1-unit increase in public pension entitlement is estimated to reduce unmet medical need by 1.65 percentage points (95% CI −1.19 to −2.10).

**Figure 1:**
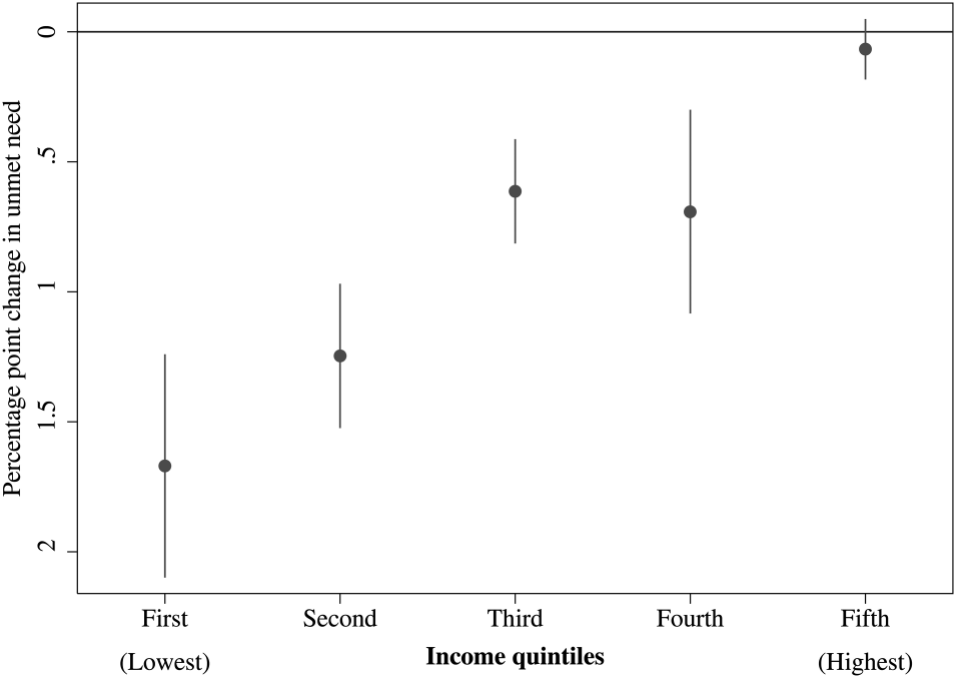
Increased public pension entitlement and unmet medical need due to cost by income distribution, 2004–2010.

If the benefits of public pension entitlement are greatest in older persons in the poorest income quintile, then it would be expected that public pension entitlement may reduce inequalities in access to healthcare. To test this, we estimate the association between public pension entitlement and unmet medical need (specifically due to cost) in the remaining four income quintiles. Web appendix 3 reports the estimates for all of these five separate models. In contrast to the poorest income quintile, the association between public pension entitlement and unmet medical need due to cost in the wealthiest quintile is not significantly different from zero (0.03, 95% CI −0.16 to 0.21). As shown in figure 1, the association between public pension entitlement and reductions in unmet medical need becomes progressively attenuated at higher points in the income distribution so that, at the top quintile, there is no significant relationship (p*=*0.73; figure 1).

**Figure 2:**
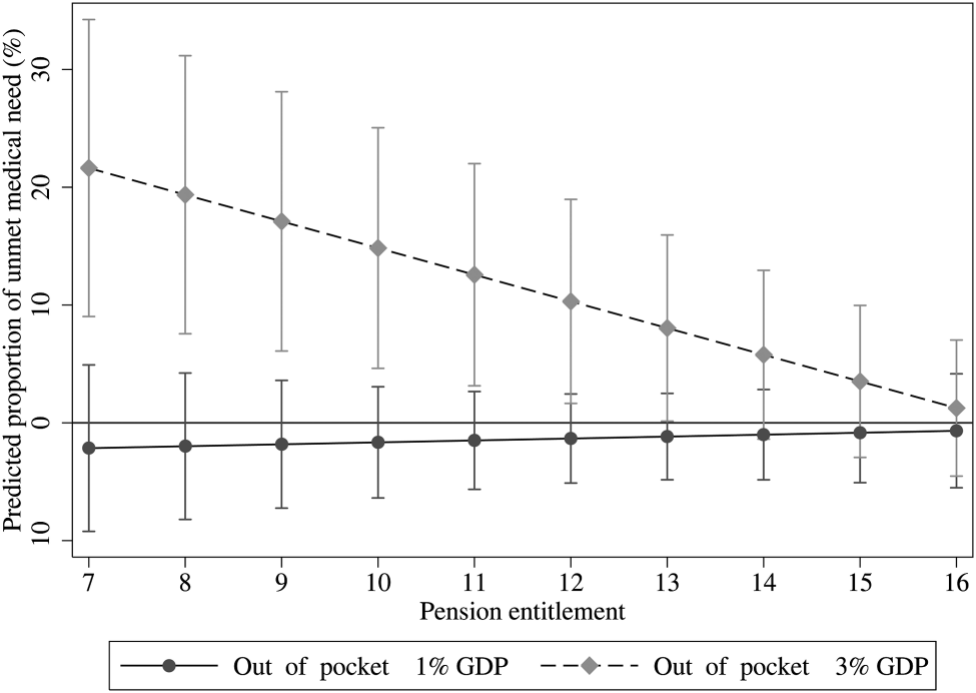
Association between unmet medical need and the level of out-of-pocket spending on healthcare as a proportion of GDP, 2004–2010. GDP, gross domestic product.

One mechanism linking public pension entitlement and unmet medical need is the financial cost of accessing healthcare, which is mitigated by generous public pensions. As noted above, out-of-pocket payments for healthcare, such as user charges, vary between countries and are one measure of the extent to which the health system is commodified, that is, the degree to which health services are treated as any other commodity to be traded or paid for on the open market. Given cross-national variation in the commodification of healthcare, we anticipate that the influence of public pension entitlement on unmet medical need would be greatest in countries where out-of-pocket payments are high. Out-of-pocket expenditure in this instance is any cost sharing with private or social health insurance. It does not include direct payment to corporations and therefore predominantly captures the amount spent on user charges, deductibles and patient's direct payments. To test this mechanism, we re-estimate our model but include an interaction term between public pension entitlement and out-of-pocket payments as a proportion of GDP. The average level of out-of-pocket expenditure as a proportion of GDP was 1.69%, the minimum was 0.63% and the maximum was 3.49%. We find that public pension entitlement mitigates the influence of out-of-pocket payments on unmet medical need due to cost (β=−1.21 percentage points, 95% CI −0.37 to −2.06) (figure 2). In contrast, if we re-estimate this interaction between out-of-pocket payments and public pension entitlements among the working-age population (ages 16–64), there is no longer a clear association between these variables and unmet medical need (p=0.13), giving our findings greater specificity. In short, public pensions reduce unmet medical need but only in countries with high levels of out-of-pocket payments (3% of GDP), whereas in countries where out-of-pocket payments are low (1% of GDP) they have no effect.

### Does greater public pension entitlement have spillover effects for other age groups?

Pensioners may share their income with non-pensioners because they are married to or living with people who are working-age non-pensioners. Previous work finds that pension generosity improves the health of children in the same household.^13^ To examine whether public pension entitlement has any spillover effects for those not yet 65, we examined the association between increased welfare entitlement (including public pension, sickness and unemployment), economic growth and government health spending on unmet medical needs for those aged 16–64 (table 2). A 1-unit increase in public pension entitlement (the equivalent of a 10 percentage-point increase in the minimum income replacement rate) is associated with a 0.56 percentage-point decline in unmet medical need due to cost for these younger age groups (95% CI −0.043 to −1.07). As expected, this association is less than the observed relationship among those over 65 alone (see table 1). Welfare entitlement for those receiving sickness and disability support (p=0.63) or unemployment benefit (p=0.71) had no association with unmet medical need. Similarly, there was no clear relationship between government health spending on health and unmet medical need (p=0.45). A purchasing power parity (PPP)$100 increase in GDP per capita was associated with a 0.022 percentage-point decline in unmet medical need (95% CI 0.0032 to −0.048); suggesting growth in real incomes may reduce barriers to accessing healthcare for these younger age groups. Again, our measure of private pensions is not associated with changes in unmet medical need (p=0.59).

**Table 2:** Impact of public pension entitlement on unmet medical need due to cost for those aged 16–64 years, 2004–2010

	Unmet medical need due to cost (percentage point), aged 16–64					
Covariates	(Model 1)	(Model 2)	(Model 3)	(Model 4)	(Model 5)	(Model 6)
Public pension entitlement	−0.50* (−0.99 to −0.016)	−0.55* (−0.99 to −0.10)	−0.50* (−0.98 to −0.018)	−0.49* (−0.97 to −0.016)	−0.55* (−1.06 to −0.039)	−0.56* (−1.07 to −0.043)
Sickness insurance entitlement		−0.44 (−1.46 to 0.58)	−0.49 (−1.54 to 0.56)	−0.26 (−1.32 to 0.80)	0.080 (−1.09 to 1.25)	0.082 (−1.09 to 1.26)
Unemployment insurance entitlement		0.15 (−0.31 to 0.61)	0.12 (−0.36 to 0.59)	0.087 (−0.38 to 0.55)	−0.21 (−0.80 to 0.39)	−0.10 (−0.81 to 0.60)
US$100 increase in public health expenditure per capita			−0.033 (−0.17 to 0.10)	0.062 (−0.10 to 0.23)	0.047 (−0.13 to 0.23)	0.029 (−0.16 to 0.22)
US$100 increase in GDP per capita				−0.022 (−0.043 to 0.000)	−0.024 (−0.049 to 0.00071)	−0.022 (−0.048 to 0.0032)
Proportion of those aged 16–64 years with a chronic illness (%)					0.11 (−0.017 to 0.24)	0.10 (−0.027 to 0.24)
Private pension expenditure (% GDP)						0.28 (−0.77 to 1.34)
Observations	103	103	103	103	94	94
R^2^	0.058	0.068	0.071	0.11	0.15	0.15

Sources: Comparative Welfare Entitlements Dataset, Organisation for Economic Co-operation and Development (OECD) and Eurostat. Expenditure measures are adjusted for inflation and purchasing power parity. All models adjust for country-specific differences that are constant over time. 95% CIs in parentheses.

*p<0.05; **p<0.01.

GDP, gross domestic product.

### Sensitivity analyses

As a falsification test, we evaluated outcomes which would not plausibly be linked with public pension entitlement. Specifically, we would not anticipate an association of public pension entitlement with unmet medical needs due to non-financial constraints. For example, while public pensions might reduce unmet need due to cost, it should be largely unrelated to non-financial reasons for unmet medical need. As expected, we find no association between public pensions and unmet medical need due to non-financial reasons (web appendix 4). Although the replacement rates are calculated for the average wage of a production worker (a fictitious baseline for different household types), we also adjust for changes in this average that might alter the relative level of public pension entitlement. After adjusting for changes in the average production worker wage, we find that our results remain substantively unchanged (web appendix 5). To account for any secular trends in unmet medical need, we include time dummies in the model, finding that our main results do not change (web appendix 6). Changes in healthcare infrastructure may account for some of the changes in unmet medical need and so we include the number of hospital beds per 100,000 inhabitants into our models. We find that the association between public pension generosity and unmet medical need remains stable (web appendix 7). Our aggregate-level models do not include individual covariates, such age, sex, marital status, the presence of chronic illness. We re-estimate our models using a multilevel modelling approach (incorporating individual- and country-level variables) and continue to find that—as expected—greater public pension entitlements are associated with lower unmet medical need (web appendix 8). Further, the benefits of these entitlements remain concentrated among the poorest groups in these multilevel models (web appendix 9), which is consistent with web appendix 2. This also addresses the problem of changing sample sizes observed in those models, because there are no missing data at the country level for the results in web appendix 9.

## Discussion

Our study highlights three important findings. First, greater public pension entitlement is associated with reduced unmet medical need, especially among older people. Second, this association is only observed in countries with high levels of out-of-pocket expenditure on health. Third, the association between public pension entitlement and unmet need is greatest among those in the poorest income quintiles, without harming access to healthcare in the wealthiest quintiles. Even a 1% increase in the minimum income replacement rate for public pensions would reduce unmet medical need by ∼0.2% among older people who are income poor. To put these findings into perspective, take Germany and the Netherlands as two examples. Germany has a higher rate of out-of-pocket payments than the Netherlands (1.4% of GDP compared to 0.6% of GDP, respectively). Yet, if Germany had the same public pension entitlement as the Netherlands, our model predicts that Germany would reduce unmet medical need among the poorest income quintile from 2.2% to 0.2%, thereby eliminating income inequalities in unmet medical need among older people. Additionally, our models also predict that Germany would reduce the proportion of unmet medical need in the whole population to the level of the Netherlands (from 1.6% to 0.5%).

This paper explores unmet medical need among the elderly but unmet medical need has also been rising among the working-age population. Our results suggest that unemployment insurance and sickness insurance are not associated with unmet medical need among the working-age population, potentially because the unemployed and those unable to work may be exempt from co-payments or other costs associated with accessing healthcare. However, these results are suggestive only and more research is needed on this question; and we plan to examine this in more detail in future papers.

There are important limitations to this study. Given the small number of countries, we have estimated country-level, fixed-effects regression models, but our results are stable even when we use multilevel models with individual-level and country-level predictors.^33^ However, because we rely on ecological public pension entitlement data, we cannot test whether those with greater state pension entitlement are also those experiencing reduced unmet medical need. Despite this, our results have a high degree of specificity in terms of age, income, and the reason why these medical needs are unmet. Additionally, public pension entitlement is a composite measure which incorporates various policies. Changes on any of these dimensions could therefore explain why certain countries became more or less generous in a specific year. Given this variation, it is difficult to identify those policy changes that have been most closely associated with unmet medical need. However, throughout this period, almost all of the variation in public pensions is attributable to the level of minimum replacement rates, that is, the degree of income replacement offered by pension programmes, suggesting financial mechanisms are driving these associations. Our measure of public pension entitlement only captures the effect of state pensions and does not incorporate the role of occupational pensions on income, which is substantial in many European countries. However, examining the influence of changes to public pensions allows us to more clearly identify the effect of a change in income for those most at risk of unmet medical need; because groups at the bottom of the income distribution are most likely to experience unmet medical need and rely most heavily on public pensions. Our panel of countries is relatively small, but even in these circumstances (eg, when the number of countries >10 and the number time points >5) regression models that account for country-specific difference which are constant over time (so-called ‘fixed-effects’ models) are still preferable over models which do not account for these differences.^34^ Our measure of private pensions is imperfect and more work is needed to address the role of private pension provision on access to healthcare, especially given mixed results in the previous literature.^10^ ^13^ Notwithstanding the measurement error in this indicator, we find that public pension entitlements remain closely associated with access to healthcare even when we adjust for private pension provision. Finally, this self-reported measure of unmet medical need potentially obscures whether these are ‘needs’ or ‘demands’. To partially address changes in demand, we adjust for the proportion of chronically ill people aged 65 or over.

These findings have important policy implications. Many European countries have reduced public pension entitlement since the Great Recession^9^ leading to increased unmet medical need, particularly in countries with commodified healthcare systems. In Greece, for example, the introduction of user charges coupled with public pension reform will likely exacerbate unmet medical need among pensioners and those at risk of poverty to the greatest extent.^4^ ^9^ The long-term implications of these changes for pensioners remain unclear, but previous evidence indicates increased healthcare costs and may even increase the risk of frailty and mortality.^7^ While the recession has already increased financial insecurity among older people, public pension austerity is likely to further deepen precariousness and increase unmet medical needs, particularly among the income poor.

**What is already known on this subject**

- Unmet medical need has been rising across many European countries.
- This rise has been concentrated among older people and those at the bottom of the income distribution.
- Greater public pension entitlements may reduce unmet medical need among older people by reducing old-age poverty, but this relationship remains uncertain.

**What this study adds**

- Greater public pension entitlements are associated with reduced unmet medical need among the older people.
- The association between public pension entitlements and unmet medical need is greater for those at the bottom of the income distribution than those at the top.
- The link between public pension entitlement and unmet medical need is only observed in countries in commodified health systems.
