# Supplementary material for: Public pensions and unmet medical need among older people: cross-national analysis of 16 European countries, 2004–2010
Source: J Epidemiol Community Health. 2016 Dec 13;71(2):174–80. doi: 10.1136/jech-2015-206257 (PMC5284463; doi:10.1136/jech-2015-206257)
Supplement: supplementary appendix [file jech-2015-206257supp_appendix.pdf]

Web Appendix

Web Appendix 1: Descriptive statistics, 2004-2010

Web Appendix 2: Impact of public pension entitlement on unmet medical adjusting for private pensions (including voluntary and mandatory), 2004 and 2010.

Web Appendix 3: Impact of public pension entitlement on unmet medical need due to cost across the income distribution, ages 65 or over, 2004 and 2010.

Web Appendix 4: Impact of public pension entitlement on unmet medical need due to other reason, ages 65 and over, 2004 and 2010.

Web Appendix 5: Impact of public pension entitlement on unmet medical adjusting for the average production worker wage, 2004 and 2010.

Web Appendix 6: Impact of public pension entitlement on unmet medical adjusting for time dummies, 2004 and 2010.

Web Appendix 7: Impact of public pension entitlement on unmet medical adjusting for healthcare infrastructure, 2004 and 2010.

Web Appendix 8: Linear probability model of association between public pension entitlement and unmet medical need due to cost among older people (65+), 2004 and 2010, EU-SILC.

Web Appendix 9: Association between public pension entitlement and unmet medical need due to cost by income distribution, 2004-2010, EU-SILC

Web Appendix 1: Descriptive statistics, 2004-2010

Variables	Number of observations	Mean	SD	Mean annual change	Largest annual decline	Largest annual increase
Unmet medical need (65+)	103	2.43	2.84	-0.06	-3.3	3.9
Public pension entitlement	103	12.83	1.60	0.11	-0.4	3.4
Sickness entitlement	103	11.70	2.39	-0.02	-1.6	0.6
Unemployment insurance entitlement	103	10.44	2.22	-0.05	-1.9	0.7
Public health expenditure per capita (US$100)	103	21.06	5.24	0.69	-1.86	5.99
GDP per capita (US$100)	103	302.19	57.90	4.23	-29.84	23.56
Proportion of older people with a chronic illness (%)	103	29.29	7.27	0.05	-9.7	12.2
Private pension expenditure (% GDP)	103	1.40	1.49	0.02	-0.8	1.9

Notes: Sources: Comparative Welfare Entitlements database, OECD and Eurostat.

Web Appendix 2: Impact of public pension entitlement on unmet medical adjusting for private pensions (including voluntary and mandatory), 2004 and 2010.

	Unmet medical need due to cost (percentage point) among over 65s	
**Covariates**	(1)	(2)
Public pension entitlement	-1.11**	-1.11**
	[-1.66,-0.55]	[-1.67,-0.55]
Sickness insurance entitlement	-0.35	-0.35
	[-1.59,0.88]	[-1.60,0.91]
Unemployment insurance entitlement	0.15	0.16
	[-0.41,0.72]	[-0.39,0.71]
US$100 increase in public health expenditure per capita	0.20	0.19
	[-0.067,0.46]	[-0.075,0.46]
US$100 increase in GDP per capita	-0.030	-0.030
	[-0.064,0.0039]	[-0.065,0.0054]
Proportion of older people with a chronic illness (%)	-0.022	-0.022
	[-0.11,0.067]	[-0.11,0.067]
Private pensions (% of GDP)	—	0.035
		[-0.77,0.84]
Observations	103	103
R^2	0.35	0.35

Notes: Sources: Comparative Welfare Entitlements database, OECD, and Eurostat. Expenditure measures are adjusted for inflation and purchasing-power parity. All models adjust for country-specific differences that are constant over time. 95% confidence intervals in brackets.
* $p < 0.05$, ** $p < 0.01$

Web Appendix 3: Impact of public pension entitlement on unmet medical need due to cost across the income distribution, ages 65 or over, 2004 and 2010.

	**Unmet medical need due to cost (percentage point) across income quintiles**				
**Covariates**	**First (poorest) quintile**	**Second quintile**	**Third quintile**	**Fourth quintile**	**Fifth (richest) quintile**
Public pension entitlement	-1.65** [-2.10,-1.19]	-1.22** [-1.54,-0.91]	-0.61** [-0.81,-0.40]	-0.60** [-0.84,-0.36]	0.029 [-0.16,0.21]
Sickness insurance entitlement	-0.53 [-1.96,0.89]	0.077 [-3.04,3.19]	0.056 [-0.93,1.04]	-2.46 [-6.65,1.72]	0.017 [-1.30,1.33]
Unemployment insurance entitlement	0.34 [-0.46,1.13]	0.54 [-0.21,1.29]	0.36 [-0.11,0.83]	3.47 [-0.74,7.68]	0.11 [-1.11,1.33]
US$100 increase in public health expenditure per capita	0.091 [-0.24,0.42]	0.11 [-0.19,0.40]	0.18 [-0.100,0.45]	-0.0060 [-0.21,0.20]	0.0069 [-0.071,0.085]
US$100 increase in GDP per capita	-0.029 [-0.066,0.0083]	-0.0026 [-0.043,0.038]	-0.021 [-0.053,0.011]	0.029 [-0.023,0.081]	-0.017 [-0.045,0.012]
Proportion of older people with a chronic illness (%)	0.049 [-0.13,0.23]	-0.034 [-0.21,0.15]	-0.024 [-0.092,0.044]	-0.071 [-0.16,0.021]	0.000028 [-0.079,0.079]
Observations	81	68	67	45	38
R^2	0.39	0.30	0.31	0.36	0.16

Notes: Sources: Comparative Welfare Entitlements database and Eurostat. Expenditure measures are adjusted for inflation and purchasing-power parity. All models adjust for country-specific differences that are constant over time. 95% confidence intervals in brackets. Some countries do not report unmet medical need by income quintile are those countries that report it for the poorest quintile may not report it for the richest quintile. Hence the sample size is not consistent across the income distribution.

* $p < 0.05$, ** $p < 0.01$

Web Appendix 4: Impact of public pension entitlement on unmet medical need due to other reason, ages 65 and over, 2004 and 2010.

	Unmet medical need due to other reasons (percentage point) over 65s			
Covariates	(Model 1)	(Model 2)	(Model 3)	(Model 4)
Public pension entitlement	0.079<br>[-0.23,0.39]	0.036<br>[-0.25,0.33]	-0.070<br>[-0.30,0.16]	-0.060<br>[-0.26,0.14]
Sickness insurance entitlement		0.021<br>[-0.47,0.51]	0.16<br>[-0.42,0.73]	0.37<br>[-0.26,1.01]
Unemployment insurance entitlement		0.34<br>[-0.21,0.90]	0.42<br>[-0.12,0.95]	0.38<br>[-0.086,0.86]
US$100 increase in public health expenditure per capita			0.078<br>[-0.012,0.17]	0.17*<br>[0.036,0.30]
US$100 increase in GDP per capita				-0.020**<br>[-0.033,-0.0070]
Observations	103	103	103	103
R^2	0.0043	0.081	0.12	0.24

Notes: Sources: Comparative Welfare Entitlements database and Eurostat. Expenditure measures are adjusted for inflation and purchasing-power parity. All models adjust for country-specific differences that are constant over time. 95% confidence intervals in brackets.

* $p < 0.05$, ** $p < 0.01$

Web Appendix 5: Impact of public pension entitlement on unmet medical adjusting for the average production worker wage, 2004 and 2010.

	Unmet medical need (percentage point)			
**Covariates**	**Whole population**	**Poorest income quintile**	**Aged 65 or over**	**Aged 65 or over in the poorest income quintile**
Public pension entitlement	-0.66* [-1.19,-0.14]	-1.26** [-2.10,-0.42]	-1.12** [-1.77,-0.46]	-1.58** [-2.23,-0.94]
Sickness insurance entitlement	-0.29 [-1.28,0.70]	-0.30 [-1.46,0.85]	-0.44 [-1.62,0.74]	-0.34 [-1.06,0.38]
Unemployment insurance entitlement	0.19 [-0.33,0.71]	0.54 [-0.25,1.34]	0.11 [-0.45,0.67]	0.68 [-0.43,1.79]
US$100 increase in public health expenditure per capita	0.082 [-0.098,0.26]	0.044 [-0.14,0.23]	0.18 [-0.063,0.42]	0.088 [-0.21,0.39]
US$100 increase in GDP per capita	-0.023 [-0.058,0.012]	-0.022 [-0.071,0.027]	-0.029 [-0.063,0.0049]	-0.033 [-0.084,0.018]
Increase in average production worker wage[1]	0.0093 [-0.0095,0.028]	0.021 [-0.011,0.053]	-0.00079 [-0.018,0.017]	0.037 [-0.046,0.12]
Observations	103	103	103	103
R^2	0.0043	0.081	0.12	0.24

Notes: Sources: Comparative Welfare Entitlements database and Eurostat. Expenditure measures are adjusted for inflation and purchasing-power parity. All models adjust for country-specific differences that are constant over time. 95% confidence intervals in brackets.

1 – Each unit increase represents a 1000 unit in the local currency. The average production worker wage is a fictive worker in the manufacturing sector who is 40 years old, has been working for the 20 years preceding the loss of income or the benefit period.

* $p < 0.05$, ** $p < 0.01$

Web Appendix 6: Impact of public pension entitlement on unmet medical adjusting for time dummies, 2004 and 2010.

	**Unmet medical need due to cost (percentage point) among over 65s**
**Covariates**	(Model 1)
Public pension entitlement	-0.97** [-1.49,-0.46]
Sickness insurance entitlement	-0.39 [-1.60,0.83]
Unemployment insurance entitlement	0.093 [-0.39,0.57]
US$100 increase in public health expenditure per capita	0.36* [0.048,0.66]
US$100 increase in GDP per capita	-0.047 [-0.10,0.010]
Proportion of older people with a chronic illness (%)	-0.026 [-0.097,0.046]
Time dummies	Y
Observations	103
R^2	0.39

Notes: Sources: Comparative Welfare Entitlements database and Eurostat. Expenditure measures are adjusted for inflation and purchasing-power parity. All models adjust for country-specific differences that are constant over time and for time dummies (fixed-effects). 95% confidence intervals in brackets.
* $p < 0.05$, ** $p < 0.01$

Web Appendix 7: Impact of public pension entitlement on unmet medical adjusting for healthcare infrastructure, 2004 and 2010.

	**Unmet medical need due to cost (percentage point) among over 65s**
**Covariates**	(Model 1)
Public pension entitlement	-1.14** [-1.73,-0.55]
Sickness insurance entitlement	0.068 [-1.87,2.01]
Unemployment insurance entitlement	0.13 [-0.42,0.68]
US$100 increase in public health expenditure per capita	0.20 [-0.067,0.46]
US$100 increase in GDP per capita	-0.037 [-0.079,0.0052]
Proportion of older people with a chronic illness (%)	-0.027 [-0.12,0.068]
Hospital beds per 100,000 inhabitants	-0.0047 [-0.017,0.0073]
Observations	91
R^2	0.37

Notes: Sources: Comparative Welfare Entitlements database and Eurostat. Expenditure measures are adjusted for inflation and purchasing-power parity. All models adjust for country-specific differences that are constant over time and for time dummies (fixed-effects). 95% confidence intervals in brackets.
* $p < 0.05$, ** $p < 0.01$

Web Appendix 8: Linear probability model of association between public pension entitlement and unmet medical need due to cost among older people (65+), 2004 and 2010, EU-SILC.

**Covariates**	**Unmet medical need due to cost among over 65s**
Pension entitlement	-0.0057**
	(0.00089)
Sickness insurance entitlement	-0.0021
	(0.0023)
Unemployment insurance entitlement	0.00077
	(0.0016)
US$100 increase in public health expenditure per capita	0.0014
	(0.0011)
US$100 increase in GDP per capita	-0.0026
	(0.00018)
Observations	312,095
Countries	16
Years	6

Notes: Standard errors in parentheses. Model includes country and year fixed-effects capturing between country differences that are relatively stable over time and spatial correlation across countries in a given year, such as the recession. Model also adjusts for age, sex, marital status, and the presence of a chronic illness. Robust standard errors are clustered at the country-level.

* $p < 0.05$, ** $p < 0.01$

Web Appendix 9: Association between public pension entitlement and unmet medical need due to cost by income distribution, 2004-2010, EU-SILC

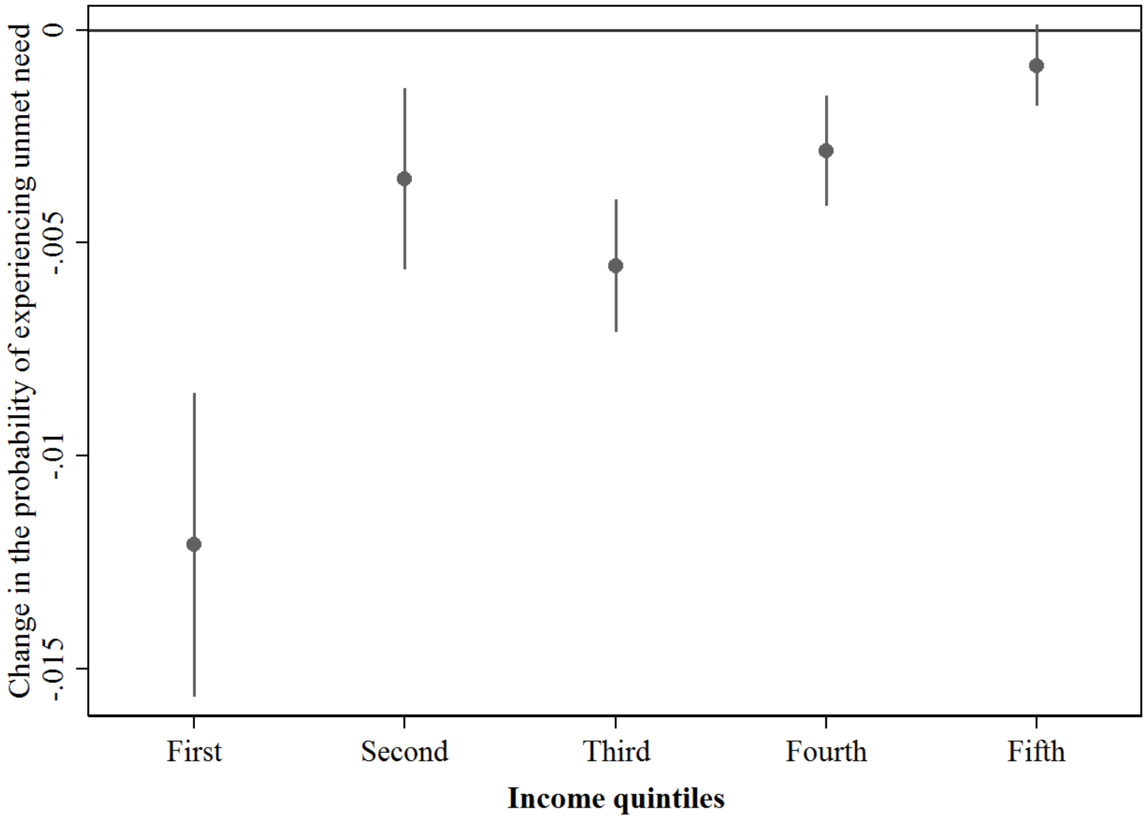

Notes: Sources: Comparative Welfare Entitlements database and Eurostat. All models adjust for sickness insurance entitlement, unemployment insurance entitlement, economic growth, government expenditure on health, age, sex, marital status, and the presence of a chronic illness. All models also adjust for country-specific differences that are constant over time and year fixed-effects. Vertical bars are 95% confidence intervals.
